# Monitoring influenza virus susceptibility to oseltamivir using a new rapid assay, iART

**DOI:** 10.2807/1560-7917.ES.2017.22.18.30529

**Published:** 2017-05-04

**Authors:** Larisa V Gubareva, Eric Fallows, Vasiliy P Mishin, Erin Hodges, Abdullah Brooks, John Barnes, Alicia M Fry, William Kramp, Roxanne Shively, David E Wentworth, Kristin Weidemaier, Ross Jacobson

**Affiliations:** 1 Influenza Division, National Center for Immunization and Respiratory Disease, Centers for Disease Control and Prevention (CDC), Atlanta, GA, United States; 2 Becton Dickinson, Research Triangle Park, North Carolina, United States; 3 Bloomberg School of Public Health, Johns Hopkins University, Baltimore, MD, United States; 4 International Centre for Diarrhoeal Disease Research, Bangladesh, Dhaka, Bangladesh; 5 Biomedical Advanced Research and Development Authority (BARDA), Washington DC, United States

**Keywords:** air-borne infections, viral infections, influenza virus, surveillance

## Abstract

A new rapid assay for detecting oseltamivir resistance in influenza virus, iART, was used to test 149 clinical specimens. Results were obtained for 132, with iART indicating 41 as ‘resistant’. For these, sequence analysis found known and suspected markers of oseltamivir resistance, while no such markers were detected for the remaining 91 samples. Viruses isolated from the 41 specimens showed reduced or highly reduced inhibition by neuraminidase inhibition assay. iART may facilitate broader antiviral resistance testing.

Early detection of drug-resistant influenza viruses is needed for timely modification of policies and recommendations on the use of antivirals [1]. In many countries, neuraminidase (NA) inhibitor(s) are the medications of choice for treatment and prophylaxis of influenza infections, with oseltamivir being most commonly prescribed. The rapid, global spread of oseltamivir-resistant A(H1N1) viruses that emerged in Norway in 2008, necessitated close monitoring of oseltamivir resistance among circulating influenza viruses [2]. The emergence and subsequent seasonal circulation of the 2009 A(H1N1)pdm09 pandemic virus have further reinforced the need for enhanced surveillance. Moreover, there have been reports of locally transmitted oseltamivir-resistant A(H1N1)pdm09 viruses harbouring the NA amino acid (AA) substitution H275Y [3-5], the marker of clinically relevant resistance to oseltamivir [6,7]. Several genotypic methods (e.g. pyrosequencing) have been implemented by surveillance laboratories to screen clinical specimens for the presence of H275Y [8].

## Assays to detect oseltamivir-resistant influenza viruses

### Neuraminidase inhibition

Unlike sequence-based assays, the NA inhibition (NAI) assay enables the detection of potentially drug-resistant viruses regardless of underlying genetic change(s). It is the gold standard method for assessing susceptibility to NA inhibitors [9,10]. Interpretation of the NAI assay is based on the determined IC_50_, a drug concentration needed to inhibit 50% of the NA enzyme activity. Depending on the fold increase of IC_50_ compared with a control, results are reported as normal (NI), reduced (RI) or highly reduced (HRI) inhibition. In the absence of established laboratory correlates of clinically-relevant oseltamivir resistance, all viruses displaying RI/HRI are considered to be potentially drug resistant and as such are monitored [9,10]. Although useful, this method is labour intensive, complex, and requires propagation of the viruses in cell culture. Additionally, the viral NA sequence from both the isolate and matching clinical specimen should be compared to rule out culturing artefacts [9-11]. Due to the complexity of the assay and data interpretation, testing is mainly performed by specialised surveillance laboratories [10,12,13].

### New rapid prototype assay

In this study, we investigated whether the influenza Antiviral Resistance Test (iART), a rapid prototype assay developed by Becton Dickinson R and D for research use only, could be used to improve oseltamivir resistance surveillance by providing a simpler and faster testing method. iART utilises an advanced enzyme substrate that enables measurement of NA activity in virus isolates and in clinical specimens. Unlike the substrate used in the bioluminescence-based assay [14], the substrate used in iART is specific to influenza NA, making it more suitable for testing clinical specimens that may contain other pathogens. In this assay, the sample is divided between two wells of a disposable (Figure), one well containing substrate and the other well containing substrate and oseltamivir carboxylate. A simple device is used to measure the chemiluminescent signal generated from each well of the disposable. The built-in software calculates the ratio of signal intensity between the wells (R-factor), which appears on the device’s display along with the final result: ‘*resistant*’ or ‘*nonresistant’*. The threshold between *nonresistant* and *resistant* is different for type A and type B viruses, with R-factors of 0.7 and 2.2, respectively. If the NA activity is too low or absent, the message ‘*insufficient signal’* appears on the display.

**Figure:**
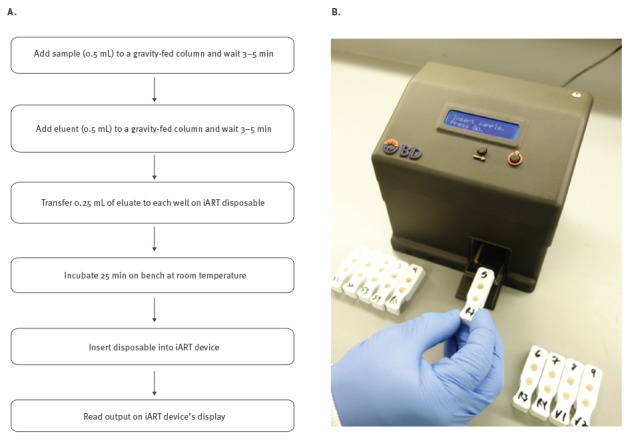
(A) Workflow of iART testing; (B) Prototype device and kit

iART: influenza Antiviral Resistance Test; VTM: viral transport medium.

Respiratory clinical specimens were stored in VTM before testing. The room temperature was monitored throughout the study and was consistently between 21 and 22˚C.

Clinical specimens (n=149) were either applied to the gravity-fed column as is, or were diluted fivefold using viral transport medium (VTM). Virus isolates (n=76) were diluted 100- or 1,000-fold using VTM to meet the assay requirement (40,000 < signal < 6,000,000 luminescent units).

## Testing viral isolates using the influenza Antiviral Resistance Test 

### International reference panel for neuraminidase inhibition assay

In the first experiment, the international reference panel for NAI assay was tested using iART and the United States Centers for Disease Control and Prevention (US CDC) standardised fluorescence-based NAI assay [13] (Table 1). Viruses identified as *resistant* by iART, displayed RI or HRI by NAI assay; viruses with NI were identified as *nonresistant*, indicating good agreement between the two assays (Table 1).

**Table 1:** Results of testing viruses from the international reference panel, for resistance to oseltamivir, using the neuraminidase inhibition (NAI) and influenza Antiviral Resistance Test (iART) assays**^a^** (n=8)

Virus	Subtype or Lineage	NA amino acid substitution^b^		NAI assay^c^		iART	
		Straight numbering	N2 numbering	IC_50_, nM (fold)^d^	Interpretation^e^	R-factor^f^	Result^g^
A/Mississippi/03/2001	H1N1	None	None	0.39 ± 0.05 (1)	NI	0.13 ± 0.04	*Nonresistant*
A/Mississippi/03/2001	H1N1	H275Y	H274Y	337.0 ± 28.93 (876)	HRI	6.06 ± 0.16	*Resistant*
A/Perth/265/2009	H1N1pdm09	None	None	0.25 ± 0.03 (1)	NI	0.12 ± 0.01	*Nonresistant*
A/Perth/261/2009	H1N1pdm09	H275Y	H274Y	171.81 ± 20.66 (1,010)	HRI	4.83 ± 0.35	*Resistant*
A/Fukui/20/2004	H3N2	None	None	0.12 ± 0.02 (1)	NI	0.16 ± 0.05	*Nonresistant*
A/Fukui/45/2004	H3N2	E119V	E119V	49.53 ± 3.89 (450)	HRI	1.01 ± 0.04	*Resistant*
B/Perth/211/2001	Yamagata	None	None	15.38 ± 0.98 (1)	NI	1.63 ± 0.14	*Nonresistant*
B/Perth/211/2001	Yamagata	D197E	D198E	98.08 ± 20.21 (6)	RI	3.13 ± 0.10	*Resistant*

IC_50_: inhibitory concentration 50%; NA: neuraminidase; R-factor: ratio of chemiluminescent signal intensity generated by viral NA activity on the substrate with and without inhibitor (i.e. oseltamivir carboxylate).

^a^ International Society for Influenza and other Respiratory Virus Diseases Antiviral Group (ISIRV AVG) NAI susceptibility reference panel, a panel of sensitive, resistant and potentially resistant reference viruses to be used as controls for the harmonisation of NAI assays (https://isirv.org/site/index.php/reference-panel).

^b^ NA amino acid substitution position is shown using both straight numbering (type/subtype specific) and N2 subtype numbering.

^c^ Tested using the United States Centers for Disease Control and Prevention (CDC) standardised fluorescence-based NAI Assay [13].

^d^ IC_50,_ drug concentration required to inhibit 50% of NA activity; mean and standard deviation of at least three independent experiments; Fold, a fold increase in IC_50_ value compared with the control (IC_50_ value for the virus lacking the amino acid substitution).

**^e^** Criteria for reporting NAI assay results based on IC_50_ fold increase compared with the reference IC_50_ value (control virus): for influenza A, normal (< 10-fold), reduced (10–100-fold) and highly reduced (> 100-fold) inhibition, and for influenza B the same criteria, but using < 5-fold, 5–50-fold and > 50-fold increases [9]; NI, normal inhibition; RI, reduced inhibition; HRI, highly reduced inhibition.

^f^ Mean and standard deviation of R-factors; results of at least three independent experiments.

^g^ Output result as shown on the device’s display; result is based on the pre-set cutoffs for influenza A (≥ 0.7) and B (≥ 2.2) viruses.

### A(H1N1)pdm09 virus isolates carrying H275Y mutations

Monitoring the spread of A(H1N1)pdm09 viruses exhibiting HRI by oseltamivir and carrying H275Y is a priority for surveillance. To evaluate the ability of iART to detect oseltamivir-resistance conferred by H275Y, 13 virus isolates with this mutation, which had been collected between 2009 and 2016 were tested. All these H275Y viruses exhibited HRI by NAI assay and were also identified as *resistant* by iART with R-factor of 5.3 ± 0.76 (Table 2).

**Table 2:** Results from neuraminidase inhibition (NAI) and iART assays for virus isolates carrying NA amino acid mutations conferring various degrees of oseltamivir resistance (n = 42) or no such mutations (controls; n= 4)

Virus	NA mutations		NAI assay			iART	
	Straight numbering	N2 numbering	IC_50_, nM Mean ± SD^a^	Fold	Interpretation^b^	R-factor, Mean ± SD	Result^c^
**A(H1N1)pdm09**							
A/Washington/29/2009	H275Y	H274Y	208.76 ± 27.05	1,228	HRI	4.1 ± 0.12	*Resistant*
A/North Carolina/39/2009	H275Y	H274Y	199.43 ± 4.38	1,173	HRI	5.17 ± 0.12	*Resistant*
A/India/1027/2013	H275Y	H274Y	185.44 ± 15.95	1,091	HRI	5.30 ± 0.38	*Resistant*
A/Delaware/08/2011	H275Y	H274Y	174.53 ± 21.24	1,027	HRI	4.16 ± 0.73	*Resistant*
A/Hawaii/67/2014	H275Y	H274Y	171.48 ± 31.01	1,009	HRI	4.21 ± 0.58	*Resistant*
A/Michigan/65/2015	H275Y	H274Y	158.76 ± 28.68	934	HRI	5.77 ± 0.22	*Resistant*
A/Denmark/528/2009	H275Y	H274Y	153.26 ± 14.47	902	HRI	5.30 ± 0.26	*Resistant*
A/Georgia/31/2016	H275Y	H274Y	150.48 ± 24.48	885	HRI	5.60 ± 0.19	*Resistant*
A/Maryland/04/2011	H275Y	H274Y	145.64 ± 4.41	857	HRI	5.30 ± 0.53	*Resistant*
A/Washington/31/2016	H275Y	H274Y	141.00 ± 9.58	829	HRI	5.76 ± 0.60	*Resistant*
A/Texas/23/2012	H275Y	H274Y	145.07 ± 30.07	805	HRI	5.48 ± 0.67	*Resistant*
A/Colorado/30/2015	H275Y	H274Y	132.40 ± 32.65	779	HRI	5.84 ± 0.30	*Resistant*
A/Texas/48/2009	H275Y	H274Y	120.22 ± 18.65	707	HRI	3.85 ± 0.19	*Resistant*
A/Bolivia/1278/2014	I223R	I222R	11.68 ± 0.15	65	RI	1.99 ± 0.30	*Resistant*
A/Tennessee/24/2016	S247R	S246R	6.61 ± 0.45	37	RI	5.67 ± 0.47	*Resistant*
A/India/1819/2016	S247R	S246R	6.31 ± 0.09	35	RI	7.58 ± 0.47	*Resistant*
A/Dnipro/133/2014	S247R	S246R	5.66 ± 0.10	31	RI	3.79 ± 0.35	*Resistant*
A/Chile/1579/2009	I223K	I222K	2.84 ± 0.65	16	RI	0.42 ± 0.03	*Nonresistant*
A/Pennsylvania/05/2016	D199G	D198G	1.47 ± 0.03	8	NI	1.03 ± 0.26	*Resistant*
A/California/12/2012	Control^d^		0.18 ± 0.06	1	NI	0.24 ± 0.16	*Nonresistant*
**A(H3N2)**							
A/Bethesda/956/2006	R292K	R292K	> 1,000	> 14,285	HRI	7.22 ± 0.24	*Resistant*
A/Texas/12/2007	E119V	E119V	37.92 ± 5.56	542	HRI	1.06 ± 0.11	*Resistant*
A/Massachusetts/07/2013	E119V	E119V	37.33 ± 10.40	533	HRI	1.04 ± 0.04	*Resistant*
A/Arkansas/13/2013	E119V	E119V	34.88 ± 2.69	498	HRI	1.22 ± 0.11	*Resistant*
A/Illinois/03/2015	E119V	E119V	31.98 ± 3.70	458	HRI	1.32 ± 0.14	*Resistant*
A/Washington/33/2014	E119V	E119V	29.83 ± 6.56	426	HRI	1.19 ± 0.08	*Resistant*
A/Massachusetts/07/2013	Del245–248	Del245–248	21.70 ± 3.59	310	HRI	1.74 ± 0.06	*Resistant*
A/Washington/01/2007	Control		0.07 ± 0.02	1	NI	0.16 ± 0.07	*Nonresistant*
**B/Victoria lineage**							
B/Florida/103/2016	A200T	A201T	318.19 ± 37.76	23	RI	7.30 ± 0.09	*Resistant*
B/Bangladesh/3008/2013	E117G	E119G	115.54 ± 10.19	8	RI	4.34 ± 0.45	*Resistant*
B/Laos/1471/2016	N294S	N294S	108.37 ± 12.31	8	RI	2.29 ± 0.55	*Resistant*
B/Mexico/4260/2016	I221V	I222V	58.57 ± 9.38	4	NI	2.42 ± 0.03	*Resistant*
B/Laos/0425/2016	Control		13.99 ± 0.61	1	NI	0.95 ± 0.18	*Nonresistant*
**B/Yamagata lineage**							
B/Illinois/03/2008	E117A	E119A	> 1,000	> 112	HRI	10.44 ± 0.26	*Resistant*
B/Hong Kong/36/2005	R374K	R371K	> 1,000	> 112	HRI	9.11 ± 0.28	*Resistant*
B/Memphis/20/1996	R150K	R152K	591.47 ± 61.79	66	HRI	3.99 ± 0.36	*Resistant*
B/Vermont/15/2015	D197N	D198N	73.76 ± 8.17	8	RI	2.39 ± 0.18	*Resistant*
B/Santiago/75552/2015	D197N	D198N	54.81 ± 6.48	6	RI	2.59 ± 0.24	*Resistant*
B/Gorbea/75877/2015	D197N	D198N	49.51 ± 8.85	6	RI	2.49 ± 0.03	*Resistant*
B/Ontario/1110/2011	H273Y	H274Y	57.48 ± 6.98	6	RI	1.66 ± 0.16	*Nonresistant*
B/California/88/2015	H273Y	H274Y	50.18 ± 7.58	6	RI	1.78 ± 0.34	*Nonresistant*
B/Florida/05/2016	K152N	K154N	43.59 ± 4.88	5	RI	4.09 ± 0.29	*Resistant*
B/Utah/15/2016	D197N	D198N	38.72 ± 3.19	4	NI	3.06 ± 0.58	*Resistant*
B/Rochester/02/2001	D197N	D198N	37.08 ± 1.96	4	NI	2.40 ± 0.33	*Resistant^e^*
B/Wisconsin/42/2016	G407S	G402S	36.08 ± 3.52	4	NI	1.99 ± 0.10	*Nonresistant*
B/Rochester/02/2001	Control		8.93 ± 0.82	1	NI	0.97 ± 0.12	*Nonresistant*

Del: deletion; iART: influenza Antiviral Resistance Test; IC_50_: inhibitory concentration 50%; R-factor: ratio of chemiluminescent signal intensity generated by viral neuraminidase activity on the substrate, with and without inhibitor (i.e. oseltamivir carboxylate); SD: standard deviation.

^a^ Mean and standard deviation based on the results from at least three independent experiments.

^b^ Criteria for reporting NAI assay results based on an IC_50_ fold increase compared with the reference IC_50_ value (control virus): for influenza A, normal (< 10-fold), reduced (10–100-fold) and highly reduced (> 100-fold) inhibition, and for influenza B the same criteria, but using < 5-fold, 5–50-fold and > 50-fold increases [9]; NI, normal inhibition; RI, reduced inhibition; HRI, highly reduced inhibition.

^c^ Output result as shown on the device’s display; result is based on the pre-set cutoffs for influenza A (≥ 0.7) and B (≥ 2.2) viruses.

^d^ Control, a virus lacking NA changes (amino acid substitutions or deletions) associated with altered inhibition by oseltamivir, was included for each antigenic group (type/subtype/lineage) and used to determine a fold change and a degree of inhibition.

^e^ Two results were displayed as *resistant* and one as *nonresistant*.

### Virus isolates containing a mix of influenza viruses with and without H275Y mutations

In some instances, a sample may contain the drug-resistant and wild-type viruses (mix), but still be detected as normally inhibited in the NAI assay. To assess the ability of iART to detect oseltamivir resistance in such samples, we next tested samples with increasing proportions of H275Y (as determined by pyrosequencing [15]). Notably, isolates containing ≥ 24% of the H275Y variant were identified as *resistant* by iART, whereas NAI assay required ≥ 52% of the H275Y variant to detect RI, suggesting that iART was more efficient at this task (Table 3).

**Table 3:** Results from neuraminidase inhibition (NAI) and iART assays on mixtures of influenza A(H1N1)pdm09 viruses containing different proportions of mutants with H275Y in the neuraminidase (n = 22)

Virus	Pyrosequencing (%)^a^		NAI assay		iART	
	H275	H275Y	IC_50_, nM (Fold)^b^	Interpretation^c^	R-factor	Result^d^
A/Louisiana/08/2013	0	100	190.84 (1,004)	HRI	5.97	*Resistant*
A/Mississippi/11/2013	3	97	177.62 (935)	HRI	6.67	*Resistant*
A/North Carolina/04/2014	3	97	199.91 (1,052)	HRI	6.17	*Resistant*
A/Michigan/73/2016	3	97	157.39 (828)	HRI	5.89	*Resistant*
A/Texas/09/2014	7	93	131.02 (690)	HRI	5.39	*Resistant*
A/Texas/100/2013	9	91	150.21 (791)	HRI	5.03	*Resistant*
A/Massachusetts/06/2016	10	90	121.85 (641)	HRI	6.03	*Resistant*
A/Pennsylvania/18/2014	11	89	127.1 (669)	HRI	6.20	*Resistant*
A/Florida/10/2014	14	86	111.35 (586)	HRI	6.46	*Resistant*
A/Colorado/07/2014	16	84	110.24 (580)	HRI	6.22	*Resistant*
A/Brazil/0257 S2/2016	25	75	97.73 (514)	HRI	4.92	*Resistant*
A/Brazil/9061/2014	32	68	39.32 (207)	HRI	3.47	*Resistant*
A/Quebec/RV1424/2016	48	52	4.14 (22)	RI	1.93	*Resistant*
Mix #1^e^	63	37	1.37 (8)	NI	1.09	*Resistant*
A/Utah/10/2013	68	32	0.98 (5)	NI	1.25	*Resistant*
A/North Carolina/21/2013	72	28	0.95 (5)	NI	1.28	*Resistant*
Mix #2	76	24	0.73 (4)	NI	0.71	*Resistant*
Mix #3	84	16	0.49 (3)	NI	0.46	*Nonresistant*
A/Michigan/36/2016	89	11	0.57 (3)	NI	0.43	*Nonresistant*
Mix #4	92	8	0.37 (2)	NI	0.28	*Nonresistant*
Mix #5	96	4	0.35 (2)	NI	0.12	*Nonresistant*
A/Maryland/08/2013	100	0	0.22 (1)	NI	0.18	*Nonresistant*

iART: influenza Antiviral Resistance Test; IC_50_: inhibitory concentration 50%; R-factor: ratio of chemiluminescent signal intensity generated by viral neuraminidase activity on the substrate, with and without inhibitor (i.e. oseltamivir carboxylate).

^a^ Proportion of H275 and H275Y virus subpopulations was determined by a single-nt polymorphism (SNP) pyrosequencing analysis in allele quantification mode (AQ) as described in reference [15].

^b^ Fold increase calculated using the median oseltamivir IC_50_ for influenza A(H1N1)pdm09 viruses circulating during 2015/16 influenza season.

^c^ Interpretation of NAI assay results based on the fold increase in IC_50_ value: normal (< 10-fold), reduced (10–100-fold) and highly reduced (> 100-fold) inhibition; NI, normal inhibition; RI, reduced inhibition; HRI, highly reduced inhibition.

^d^ Output result as shown on the device’s display; result is based on the pre-set cutoffs for influenza A (≥ 0.7) and influenza B (≥ 2.2) viruses.

^e^ H275 and H275Y mixes were prepared by combining the two virus isolates A/Maryland/08/2013 and A/Louisiana/08/2013, at different ratios.

### Influenza virus isolates with mutations other than H275Y

Next, we assessed iART ability to detect influenza viruses harbouring NA mutations other than H275Y and displaying RI/HRI against oseltamivir (Table 2): A(H1N1)pdm09 viruses that displayed RI by oseltamivir carrying the S247R (n = 3) or I223R (n = 1) were identified as *resistant* with high R-factors for S247R and an R-factor of 1.99 ± 0.30 for I223R. One virus carrying I223K was detected as *nonresistant* with an R-factor substantially below the resistance threshold (0.42 ± 0.03). The virus carrying D199G displayed NI (eightfold) by NAI assay and was identified as *resistant* by iART (Table 2). A(H3N2) viruses that display HRI by NAI assay were all identified as *resistant* by iART. The R-factor of the R292K virus was much greater than those harbouring either E119V or a four-amino acid deletion (del245–248). Three B/Victoria/2/87-lineage viruses – harbouring E117G, N294S or A200T – that displayed RI against oseltamivir were all identified as *resistant* by iART (Table 2). B/Yamagata/16/98-lineage viruses harbouring E117A, R150K or R374K, that displayed HRI by NAI assay were identified as *resistant*; and two viruses carrying H273Y and one carrying G407 presenting borderline NI/RI were identified as *nonresistant* by iART (Table 2). Finally, a group of viruses from both B/lineages – carrying D197N, K152N and I221V – showed borderline NI/RI by NAI assay (4–8-fold), and these viruses were identified as *resistant* by iART. These results demonstrate that iART may detect some influenza viruses harbouring NA changes in the enzyme active site (e.g. D199G in A(H1N1)pdm09 and I221V in type B) that otherwise would be classified as NI by oseltamivir using NAI assay. Notably, the criteria to separate viruses exhibiting NI from those with RI is arbitrary [9], and can be refined as more data become available. The interpretation of results obtained for viruses displaying borderline IC_50_ should be made cautiously.

## Testing of clinical specimens 

Because iART was designed to detect oseltamivir-resistant viruses in human respiratory specimens, we next tested a set of 64 well-characterised specimens collected during a clinical study conducted in 2008–2010 [16] (Table 4). All the clinical specimens containing pre-pandemic A(H1N1) viruses harbouring H275Y (n = 32) were consistently identified as *resistant* with a mean R-factor of 6.86 ± 1.31. All other specimens were identified as *nonresistant* (Table 4). As expected, specimens negative for influenza (n = 10) displayed a signal below the level of detection (data not shown). These results serve as a proof-of-principle that iART can successfully detect oseltamivir-resistant H275Y viruses directly in clinical specimens.

**Table 4:** Respiratory specimens from the clinical study on the efficacy of treatment with oseltamivir tested using iART^a^ (n = 64)

Type and subtype	Number of specimens	Ct^b^	iART/R-factor	iART/result^c^
A(H1N1) H275Y^d^	32	24.40 ± 2.63	6.8 6 ± 1.31	*Resistant*
A(H1N1)pdm09	12	21.31 ± 3.25	0.06 ± 0.02	*Nonresistant*
A(H3N2)	10	21.75 ± 2.13	0.25 ± 0.11	*Nonresistant*
B	10	24.46 ± 1.81	0.99 ± 0.10	*Nonresistant*

Ct: cycle threshold; iART: influenza Antiviral Resistance Test; R-factor: ratio of chemiluminescent signal intensity generated by viral neuraminidase activity on the substrate, with and without inhibitor (i.e. oseltamivir carboxylate).

^a^ Aliquots of leftover respiratory specimens (nasal or nasopharyngeal wash) from the clinical study [13] were used for testing by iART. Specimens were initially processed on ice in the laboratory within the study clinic. The study was reviewed and approved by the ethics and research review board of the International Centre for Diarrheal Diseases, Bangladesh (icddr,b) and the institutional review board of the United States Centers for Disease Control and Prevention (CDC). All index patients provided written informed consent. Clinical specimens were aliquoted and stored at -70 °C until testing using real-time reverse-transcriptase PCR assay (rRT-PCR), pyrosequencing (to detect neuraminidase amino acid substitutions previously associated with oseltamivir resistance), virus isolation and neuraminidase inhibition assay testing. All respective virus isolates, except those carrying H275Y, displayed normal inhibition in the NAI assay. For testing using iART, 0.5 mL of clinical specimen was used; all specimens tested underwent a single freeze/thaw cycle.

^b^ Ct value as determined using rRT-PCR assay according to the CDC protocols.

^c^ Output result as shown on the device’s display; result is based on the pre-set cutoffs for influenza A (≥ 0.7) and B (≥ 2.2) viruses.

^d^ Pre-pandemic A(H1N1) carrying H275Y, straight neuraminidase amino acid numbering.

Of note, the recommended volume for the iART test in its current configuration is 0.5 mL of sample, which is often unavailable at surveillance laboratories. Moreover, clinical specimens submitted to surveillance laboratories commonly undergo freeze-thaw cycles before testing, which adversely affect the integrity of virus particles. To address these concerns, we next tested a set of residual clinical specimens from the 2015/16 US national surveillance that were previously confirmed influenza virus positive; only 0.1 mL of each specimen was used for testing using iART. Of 85 tested, 17 samples (20%) had a signal below the limit of detection; 59 samples (69%) were identified as *nonresistant*; and nine samples (11%) as *resistant* (Table 5). These nine harboured H275Y, E119V or K152N. The matching isolates of these nine clinical specimens displayed RI/HRI in the NAI assay, while the other virus isolates showed NI.

**Table 5:** Residual clinical specimens from the 2015/16 United States national influenza surveillance tested using iART (n = 85)

Type and subtype	Number of specimens tested^a^	Number of. indeterminate^b^	Number of *nonresistant^c^*	Number of *resistant^c^*	NA mutation in resistant viruses^d^
A(H1N1)pdm09	34	9	19	6	H275Y
A(H3N2)	25	5	18	2	E119V
B	26	3	22	1	K152N
Total	85	17	59	9	Not applicable

NA: neuraminidase.

^a^ Leftovers of clinical specimens submitted for United States national virological surveillance that satisfied the following criteria: (i) NA sequencing or pyrosequencing data were available; (ii) virus was recovered in cell culture and tested using NAI assay. For testing using iART, 0.1 mL of a residual clinical specimen was combined with 0.4 mL of viral transport medium (VTM, Becton Dickinson) to bring the final volume to 0.5 mL.

^b^ Indeterminate: specimen displayed a low signal to noise ratio (SNR); insufficient NA activity for testing.

^c^ Output result as shown on the device’s display; result is based on the pre-set cutoffs for influenza A (≥ 0.7) and B (≥ 2.2) viruses.

^d^ Position of amino acid residue shown using straight NA numbering (Table 1).

## Conclusion

A limitation of this study is that the effect of viral loads in relation to the performance of iART was not investigated. As the iART detects NA activity, one challenge is the difference in NA specific activities of seasonal wild-type viruses, whereby the minimal viral load needed for the iART assay may depend on the virus type/subtype and might not be generalisable. More studies are needed to establish the type/subtype specific limit of detection. Moreover, NA mutations that confer oseltamivir resistance may or may not affect the NA specific activity, so the influence of this on viral load appropriate for the assay would also have to be investigated independently for such viruses.

Taken together, however, the data presented here show that the iART assay can become a valuable tool for surveillance laboratories. iART offers a fast mean for detecting viruses displaying RI/HRI against oseltamivir in either isolates or clinical specimens. It is a simple approach where signal measurement, data analysis and interpretation are done by a compact portable device. The assay robustness is evident from its ability to test specimens under less than optimal conditions (i.e. interference from virus transport media (VTM), multiple freeze/thaw cycles, limited volume). Although iART is not a substitute for NAI assay employed by specialised laboratories, it has great potential to enable a broader adoption of influenza antiviral resistance testing in various settings.

The prototype of the iART system tested in this study was configured by the developers for surveillance applications to detect viruses that could be identified by the gold standard NAI assay. Of note, samples collected by surveillance laboratories may be stored in a variety of storage media (e.g. VTM). To accommodate various types of sample media, the current iART workflow includes a buffer exchange to remove media components that interfere with the assay. If this assay is to be used at clinical care settings, this step is not needed, since a buffer optimised for the iART assay can be used for sample collection.

Larger studies are desirable to provide a better understanding of the performance and utility of the iART assay and to establish laboratory correlates (e.g. R-factor threshold) for clinically-relevant resistance. As iART was designed to test influenza A viruses, regardless of their antigenic subtype, the utility of this rapid test in detecting oseltamivir resistance in zoonotic influenza viruses (e.g. avian A(H7N9)) needs to be evaluated, as this would facilitate pandemic preparedness. Nonetheless, we are confident that the implementation of this assay, which is available for national public health agencies, e.g. the US CDC and application by its network of influenza surveillance laboratories, can facilitate timely detection of oseltamivir resistance emergence and spread.
